# Predicting Central Serous Chorioretinopathy Recurrence Using Machine Learning

**DOI:** 10.3389/fphys.2021.649316

**Published:** 2021-11-25

**Authors:** Fabao Xu, Cheng Wan, Lanqin Zhao, Qijing You, Yifan Xiang, Lijun Zhou, Zhongwen Li, Songjian Gong, Yi Zhu, Chuan Chen, Cong Li, Li Zhang, Chong Guo, Longhui Li, Yajun Gong, Xiayin Zhang, Kunbei Lai, Chuangxin Huang, Hongkun Zhao, Daniel Ting, Chenjin Jin, Haotian Lin

**Affiliations:** ^1^State Key Laboratory of Ophthalmology, Zhongshan Ophthalmic Center, Sun Yat-sen University, Guangzhou, China; ^2^Department of Electronical and Information Engineering, Nanjing University of Aeronautics and Astronautics, Nanjing, China; ^3^Xiamen Eye Center, Affiliated to Xiamen University, Xiamen, China; ^4^Department of Molecular and Cellular Pharmacology, University of Miami Miller School of Medicine, Miami, FL, United States; ^5^Sylvester Comprehensive Cancer Center, University of Miami Miller School of Medicine, Miami, FL, United States; ^6^Department of Ophthalmology, Tongji Medical College, The Central Hospital of Wuhan, Huazhong University of Science and Technology, Wuhan, China; ^7^Department of Ophthalmology, Singapore National Eye Center, Singapore, Singapore; ^8^Center of Precision Medicine, Sun Yat-sen University, Guangzhou, China

**Keywords:** machine learning, central serous chorioretinopathy, recurrence, optical coherence tomography, imaging features

## Abstract

**Purpose:** To predict central serous chorioretinopathy (CSC) recurrence 3 and 6 months after laser treatment by using machine learning.

**Methods:** Clinical and imaging features of 461 patients (480 eyes) with CSC were collected at Zhongshan Ophthalmic Center (ZOC) and Xiamen Eye Center (XEC). The ZOC data (416 eyes of 401 patients) were used as the training dataset and the internal test dataset, while the XEC data (64 eyes of 60 patients) were used as the external test dataset. Six different machine learning algorithms and an ensemble model were trained to predict recurrence in patients with CSC. After completing the initial detailed investigation, we designed a simplified model using only clinical data and OCT features.

**Results:** The ensemble model exhibited the best performance among the six algorithms, with accuracies of 0.941 (internal test dataset) and 0.970 (external test dataset) at 3 months and 0.903 (internal test dataset) and 1.000 (external test dataset) at 6 months. The simplified model showed a comparable level of predictive power.

**Conclusion:** Machine learning achieves high accuracies in predicting the recurrence of CSC patients. The application of an intelligent recurrence prediction model for patients with CSC can potentially facilitate recurrence factor identification and precise individualized interventions.

## SUMMARY

A solid machine learning system was successfully developed to predict recurrence in patients with central serous chorioretinopathy 6 months in advance, achieving high accuracies ranging from 0.899 to 1.000.

## Introduction

Central serous chorioretinopathy (CSC) is an idiopathic ophthalmopathy characterized by detachment of the neurosensory retina in the central macular region due to serous leakage in a defective retinal pigment epithelium (RPE) (Maruko et al., 2010; Wong et al., 2016). A population-based study in Olmsted County, MN, United States reported annual age-adjusted incidences of CSC from 1980 through 2002 of 9.9 and 1.7 per 100,000 in men and women, respectively, in a predominantly Caucasian population (van Rijssen et al., 2019). In Asian populations, however, pachychoroid diseases, such as CSC and polypodal choroidal vasculopathy (PCV), have been considered to be more prevalent than in Caucasian populations (van Rijssen et al., 2019). Although most patients have a good prognosis after prompt treatment and regular follow-ups (Manayath et al., 2018; van Rijssen et al., 2019), 12.8% of patients can develop permanent visual impairment and even progress to legal blindness due to the recurrent nature of CSC and poor management during follow-ups (Framme et al., 2015; Manayath et al., 2018; Mrejen et al., 2019; van Rijssen et al., 2019). Currently, CSC is the fourth most common non-surgical retinopathy after age-related macular degeneration (AMD), diabetic retinopathy (DR) and retinal vein occlusion (RVO) (Wang et al., 2008; Manayath et al., 2018). In contrast to the top three diseases, CSC mainly occurs in young men of working age and imposes a substantial economic and medical burden on society and families (Iacono et al., 2015; Daruich et al., 2017).

Artificial intelligence (AI) has been widely applied in the medical field, particularly in ophthalmology (Caixinha and Nunes, 2017; Zhang et al., 2021). AI has achieved excellent performance in the diagnosis, treatment, and prediction of ocular diseases, including myopia, DR and AMD (Gulshan et al., 2016; Bogunovic et al., 2017; Lin et al., 2018). As Bogunovic et al. (2017) reported the application of machine learning in predicting the prognosis of AMD, the prognosis and recurrence of CSC can be modeled using machine learning. In this study, using medical records and imaging features, we established an intelligent system to predict the recurrence of CSC at 3 and 6 months after laser treatment, which will help make personalized follow-up arrangements and reduce the vision loss caused by CSC recurrence.

## Materials and Methods

### Clinical Data and Imaging Examinations

**FIGURE 1:**
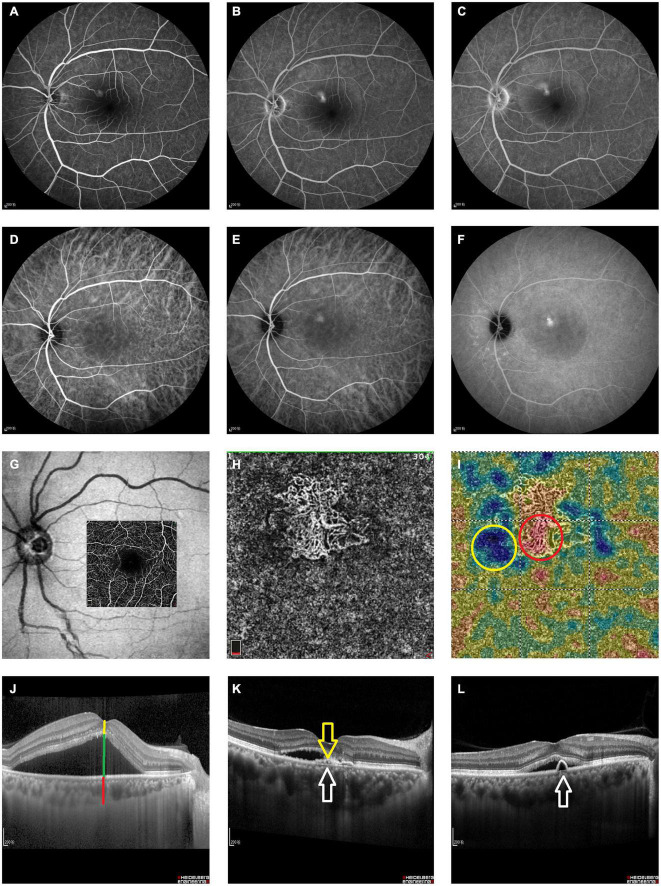
Measurement features extracted from the imaging results. All imaging features used to predict recurrence. Please see Table 1 for detailed descriptions of all measurement features. **(A–C)** Early, middle and late phase FFA of the left eye of a 52-year-old patient with CSC. **(D–F)** Contemporaneous ICGA of the same patient. **(G)** The en face projection slab area of the 3*3 pattern on OCTA. **(H)** The observation of a superficial choroidal layer on OCTA confirmed the presence of BVN. **(I)** High- and low-reflection areas in the superficial choroidal layer were surrounded with red and yellow circles, respectively. **(J)** Horizontal B-scan OCT of a patient with CSC; manual measurements were labeled as follows: yellow line, RNEL; green line, SRF; and red line, ChT; **(K)** yellow arrow, DLS; white arrow, Bruch membrane; **(L)** white arrow, PED. VA, visual acuity; FFA, fundus fluorescein angiography; ICGA, indocyanine green angiography; OCT, optical coherence tomography; OCTA, optical coherence tomography angiography; SRF, subretinal fluid; RNEL, retinal neuroepithelial layer; ChT, choroidal thickness; PED, retinal pigment epithelial detachment; DLS, double-layer sign; BVN, branching vascular network.

Both electronic medical records (20 clinical features, e.g., the duration of CSC) and images obtained using fundus fluorescein angiography (FFA), indocyanine green angiography (ICGA), optical coherence tomography angiography (OCTA), and optical coherence tomography (OCT) [145 features, e.g., double-layer sign (DLS)] were collected for analysis in Zhongshan Ophthalmic Center (ZOC) and Xiamen Eye Center (XEC) from January 2013 to September 2019 (details are provided in Supplementary Table 1 and Figure 1). The exclusion criterion was the presence of media opacities or a change in the signal strength index of the OCT images. The requirement for informed consent were waived because the study was retrospective and all images were fully anonymized. This study was conducted in accordance with the Declaration of Helsinki and supported by the ethical committee of ZOC (Ethical approval code: 2020KYPJ024).

**TABLE 1:** Patient demographics.

	**1-mo data**		**3-mo data**		**6-mo data**	
	**ZOC data**	**XEC data**	**ZOC data**	**XEC data**	**ZOC data**	**XEC data**
Patients (females)	401 (63)	60 (11)	308 (46)	30 (5)	244 (37)	19 (2)
Eyes/Recurrences	416/0	64/0	322/20	33/3	258/29	20/3
Age (Years)	43.19 ± 6.44	43.86 ± 7.06	42.87 ± 6.44	43.21 ± 7.51	42.96 ± 6.48	41.70 ± 6.73
VA (Baseline)	0.28 ± 0.21	0.29 ± 0.16	0.28 ± 0.21	0.27 ± 0.16	0.28 ± 0.22	0.28 ± 0.17
VA (Endpoint)	0.13 ± 0.16	0.11 ± 0.14	0.07 ± 0.17	0.07 ± 0.14	0.03 ± 0.17	0.04 ± 0.18

*VA, visual acuity, values are presented as the means ± standard deviations at baseline of different groups [in logarithm of minimum angle of resolution (logMAR) units]. ZOC, Zhongshan Ophthalmic Center; XEC, Xiamen Eye Center.*

Detailed information for 416 eyes of 401 patients was collected from ZOC and used for the training and internal test dataset; 64 eyes of 60 patients were collected at XEC and used for the external test dataset. Regarding the information about the types of therapy, data from ZOC included conventional laser (CL) treatment (117 eyes), subthreshold micropulse laser (SML) treatment (80 eyes), and half-dose photodynamic therapy (hd-PDT) (219 eyes); data from XEC included CL treatment (21 eyes), SML treatment (14 eyes), and hd-PDT (29 eyes). The follow-up points were 1, 3, and 6 months after the first laser treatment. Recurrence is defined as the reappearance of subretinal fluid (SRF) after having been fully absorbed on OCT B-scans.

Next, 6,732 imaging pictures (1248 FFA, 1248 ICGA, 1412 OCTA, and 2824 OCT images) were collected from ZOC, and 554 imaging pictures (192 FFA and 362 OCT images) were collected from XEC. The angiographic data from FFA and ICGA were based only on baseline data, while OCTA and OCT data were based on baseline data and longitudinal data collected at 1, 3, and 6 months after laser treatment. Measurements obtained from FFA (Heidelberg Spectralis, Heidelberg, Germany), ICGA (Heidelberg Spectralis, Heidelberg, Germany), OCTA (RTVue XR Avanti with AngioVue; Optovue Inc., Fremont, CA, United States), and OCT (Heidelberg Spectralis, Heidelberg, Germany) were extracted using Heidelberg Eye Explorer (version 1.7.1.0) and Optovue (version 2017.1.0.155) software. All features were extracted by 9 graduate students (F Xu, L Zhou, Z Li, Y Xiang, L Zhang, Y Gong, L Li, C Li, and X Zhang) and 2 senior professors (C Jin and S Gong). Detailed descriptions are provided in Supplementary Table 1.

### Data Preprocessing

Before using machine learning algorithms to predict recurrence at 3 and 6 months, we needed to preprocess the data and manage the missing data. Most of the machine learning models expect a dataset without missing values; this is difficult to achieve in real-life clinical datasets. Therefore, we centered all the other values around OCT measurements. OCT was not missing in all patients and included follow-up visits. There were only a few missing values extracted from ICGA in the ZOC dataset; we used the mean values of the corresponding features to fill in these missing values. However, in the XEC dataset, large portions of the ICGA and OCTA features were not documented. Considering the clinical significance of these features and their importance in the algorithms, we filled in these missing features by using the mean values in the full model and removed all the missing features in the simplified model. The steps of our study strictly follow the TRIPOD statement (Collins et al., 2015).

### Algorithms Used to Predict Recurrence

All our training and testing steps were performed on a workstation configured with a 32-core Intel Xeon E5 CPU with 128 GB of RAM. We used Python 3.6.8 in the Ubuntu 16.04 system with the following libraries: Jupyter (1.1.0), scikit-learn (0.19.1), and pandas (0.20.3). Six classification algorithms were trained and validated on 165 features, and they displayed state-of-the-art performance in each of the following adaptive domains: Decision Tree (Schoning and Hammann, 2018), AdaBoost.R2 (Qi et al., 2018), Gradient Boosting (Luo et al., 2017), Extreme Gradient Boosting (Ogunleye and Qing-Guo, 2019), Random Forest (Pavey et al., 2017), and Extra-Trees (Nattee et al., 2017) (details are provided in the Supplementary Materials).

We used grid search with cross-validation to select the most suitable hyperparameters for all of the algorithms described above, and the parameters and framework (Code S1) of the algorithms are listed in Supplementary Materials. After validating the results of the six original algorithms, we used the ensemble method to generate an ensemble of the best three originals and to identify a model with little bias and high robustness.

### Evaluation of the Models

The accuracies of predicting recurrence at 3 and 6 months after laser treatment were determined to evaluate the performance of the models. To predict recurrence at 3 months after treatment, we trained two models using baseline data and baseline plus 1-month data. To predict recurrence at 6 months after treatment, we trained three models using baseline data, baseline plus 1-month data, and baseline, 1-month and 3-month data. Data collected at 3 and 6 months were not used as training sets when used as outcome indicators in both models. The training data were divided into 10 subsets, and the recurrence rate of each subset was similar to that of the original data. Ten-fold cross-validation was used to evaluate the performance in prediction tasks.

### Simplified Prediction Model

To increase the accessibility of our prediction model for clinical use, we simplified the model using only the clinical data (11 clinical features, e.g., the duration of CSC) and OCT features (123 features, e.g., the DLS). The remaining features were determined according to the relative importance identified in the first round of analysis (Supplementary Figures 1–5) and the difficulty of image feature acquisition. Detailed descriptions of all remaining features are shown in Supplementary Table 2. Data preprocessing, model construction, and model evaluation were the same as described in the first round.

## Results

Four hundred sixty-one patients aged from 28 to 71 years (43.56 ± 6.64 years) were recruited in our study. The demographic information is shown in Table 1. The accuracies of predicting recurrence in all tasks with the six original algorithms and the simplified algorithms are listed in Table 2. For the six original algorithms, the ensemble algorithm achieved the best performance in predicting CSC recurrence. Therefore, all subsequent analyses were conducted solely based on the ensemble algorithm.

**TABLE 2:** Accuracy of the Recurrence Predictions in the internal test dataset and the external test dataset.

**Algorithm learner**	**3-mo (ACC, %)**		**6-mo (ACC, %)**		
**Internal test dataset**	**Baseline**	**Baseline + 1-mo**	**Baseline**	**Baseline + 1-mo**	**Baseline + 1-mo + 3-mo**
Decision tree	0.876 ± 0.017	0.916 ± 0.019	0.860 ± 0.040	0.810 ± 0.045	0.837 ± 0.037
AdaBoost	0.935 ± 0.013	0.895 ± 0.019	0.860 ± 0.019	0.845 ± 0.017	0.888 ± 0.023
Gradient boosting	0.802 ± 0.073	0.938 ± 0.016	0.907 ± 0.015	0.903 ± 0.012	0.895 ± 0.019
XGBoost	0.910 ± 0.016	0.916 ± 0.006	0.888 ± 0.022	0.899 ± 0.022	0.900 ± 0.035
Random forest	0.929 ± 0.017	0.938 ± 0.009	0.903 ± 0.017	0.888 ± 0.014	0.899 ± 0.014
Extra-trees	0.935 ± 0.010	0.935 ± 0.010	0.891 ± 0.009	0.867 ± 0.062	0.907 ± 0.015
Ensemble algorithm	0.929 ± 0.017	0.941 ± 0.011	0.903 ± 0.012	0.899 ± 0.014	0.903 ± 0.012
**External test dataset**	**Baseline**	**Baseline + 1-mo**	**Baseline**	**Baseline + 1-mo**	**Baseline + 1-mo + 3-mo**
Ensemble algorithm	0.939	0.970	0.950	0.950	1.000

**Simplified model**	**3-mo (ACC, %)**		**6-mo (ACC, %)**		
**Internal test dataset**	**Baseline**	**Baseline + 1-mo**	**Baseline**	**Baseline + 1-mo**	**Baseline + 1-mo + 3-mo**

Decision tree	0.889 ± 0.009	0.898 ± 0.016	0.822 ± 0.045	0.833 ± 0.046	0.861 ± 0.048
AdaBoost	0.913 ± 0.013	0.923 ± 0.006	0.818 ± 0.019	0.833 ± 0.027	0.880 ± 0.030
Gradient boosting	0.789 ± 0.081	0.845 ± 0.028	0.872 ± 0.024	0.884 ± 0.020	0.892 ± 0.039
XGBoost	0.913 ± 0.020	0.913 ± 0.009	0.899 ± 0.026	0.903 ± 0.027	0.896 ± 0.039
Random forest	0.929 ± 0.071	0.926 ± 0.016	0.899 ± 0.025	0.896 ± 0.022	0.888 ± 0.018
Extra-trees	0.926 ± 0.016	0.929 ± 0.019	0.899 ± 0.028	0.896 ± 0.031	0.899 ± 0.014
Ensemble algorithm	0.922 ± 0.021	0.926 ± 0.016	0.903 ± 0.024	0.899 ± 0.022	0.903 ± 0.017
**External test dataset**	**Baseline**	**Baseline + 1-mo**	**Baseline**	**Baseline + 1-mo**	**Baseline + 1-mo + 3-mo**
Ensemble algorithm	0.970	0.939	0.950	0.950	1.000

*ACC, accuracy of the recurrence prediction at 3 and 6 months after laser treatment compared with the ground truth. The results were stratified according to the follow-up period and the points input into the algorithms. The best learner in all cases was the ensemble algorithm.*

For the internal test dataset, if only baseline data were used, the accuracies of the recurrence prediction were 0.929 and 0.903 at 3 and 6 months, respectively. If the baseline data and all previous follow-up data were used, the accuracies were 0.941 and 0.903 for the 3- and 6-month predictions, respectively. For the external test dataset, if only baseline data were used, the accuracies of the recurrence prediction were 0.939 and 0.950 at 3 and 6 months, respectively. If the baseline data and all previous follow-up data were used, the accuracies were 0.970 and 1.000 for the 3- and 6-month predictions, respectively. The simplified model exhibited comparable accuracy for recurrence prediction (Table 2).

**FIGURE 2:**
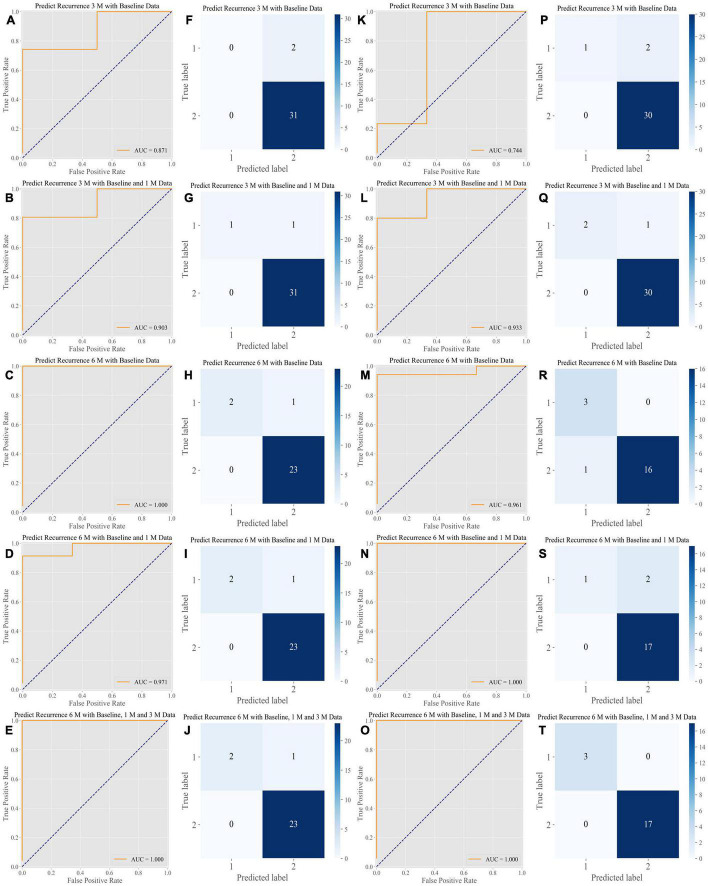
Performance of the algorithms in the internal test dataset and the External Test Dataset of the Full Model. Our algorithms presented a stable and high level of accuracy in all prediction tasks. **(A–E)** ROC analysis of the performance of the algorithms in the internal test dataset. The AUCs ranged from 0.871 to 1.000 for predictions obtained at 3 and 6 months, respectively. **(F–J)** CM of the classification provided by the algorithms in the internal test dataset. **(K–O)** ROC analysis of the performance of the algorithms in the external test dataset. The AUCs ranged from 0.744 to 1.000 for predictions at 3 and 6 months, respectively. **(P–T)** CM of the classification provided by the algorithms in the external test dataset. ROC, receiver operating characteristic curve; AUC, area under the curve; CM, confusion matrix.

**FIGURE 3:**
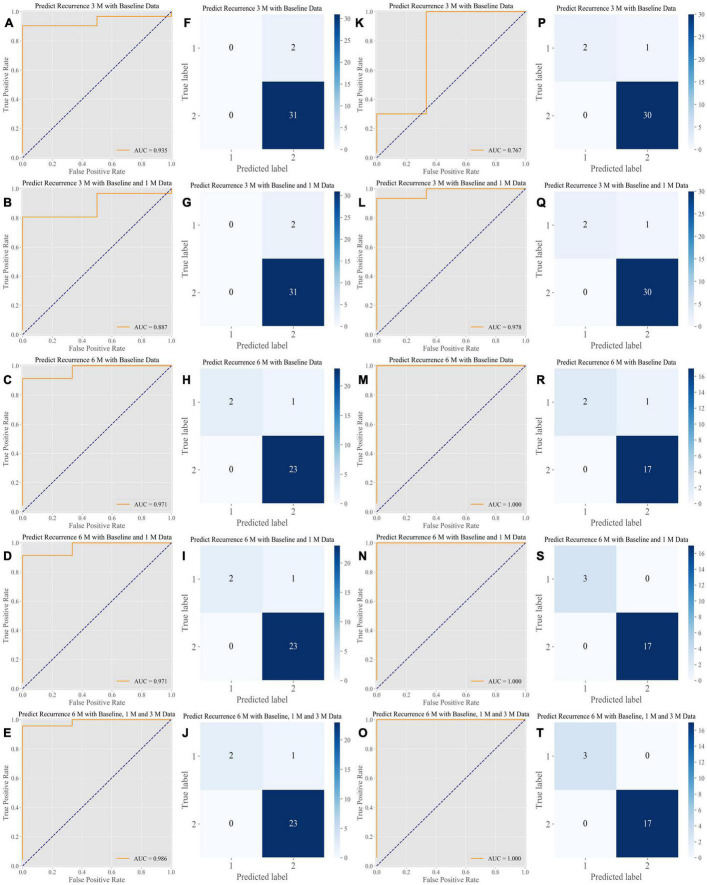
Performance of the algorithms in the internal test dataset and the External Test Dataset of the Simplified Prediction Model. The simplified prediction model shows a comparable level of predictive power. **(A–E)** ROC analysis of the performance of the algorithms in the primary validation dataset. The AUCs ranged from 0.887 to 0.986 for predictions at 3 and 6 months, respectively. **(F–J)** CM of the classification performed by the algorithms in the primary validation dataset. **(K–O)** ROC analysis of the performance of the algorithms in the external testing dataset. The AUCs ranged from 0.767 to 1.000 for predictions at 3 and 6 months, respectively. **(P–T)** CM of the classification performed by the algorithms in the external testing dataset. ROC, receiver operating characteristic curve; AUC, area under the curve; CM, confusion matrix.

The ensemble algorithm achieved highly precise predictions; the areas under the curve (AUCs) ranged from 0.871 to 0.903 at 3 months and from 0.971 to 1.000 at 6 months for the cross-validation dataset and from 0.744 to 0.933 at 3 months and 0.961 to 1.000 at 6 months for the external test dataset (Figure 2). The simplified prediction model exhibited an analogous level of precision for the predictions; the AUCs ranged from 0.887 to 0.935 at 3 months and 0.971 to 0.986 at 6 months in the cross-validation dataset and from 0.767 to 0.978 at 3 months and 1.000 at 6 months in the external test dataset (Figure 3). The distributions of the prediction results and ground truth in each task were revealed in the confusion matrix (CM) shown in Figures 2, 3. In addition, the receiver operating characteristic curve (ROC curve) and CM show that the inclusion of additional follow-up data helped obtain more accurate recurrence predictions, and the short-term prediction ability tended to outperform the long-term prediction ability. The weights of features for recurrence predictions at 3 and 6 months are shown in Supplementary Figures 1–10.

## Discussion

To our knowledge, this is the first study to establish a machine learning algorithm based on clinical medical records and imaging data for predicting the recurrence of CSC. In clinical work, treatments for CSC often lead to a satisfactory prognosis, as several methods, such as CL treatment, SML treatment and hd-PDT, have been reported to yield high SRF absorption effectiveness for 3 months (Wang et al., 2008; Iacono et al., 2015; Scholz et al., 2016). Therefore, in addition to diagnosing and applying an appropriate therapy for patients with CSC, the greater challenge for ophthalmologists is to properly manage follow-up arrangements and promptly treat the recurrence.

The machine learning model used in the present study achieves excellent performance in predicting recurrence with high accuracy. The accuracy of each prediction algorithm at the target time point is approximately 90% or higher. Based on the prediction results, ophthalmologists and general practitioners can make personalized follow-up plans to monitor recurrences. Predicting disease recurrence is crucial for a CSC patient to understand their condition. In addition, a recurrence prediction at 3 or 6 months after treatment will help doctors determine an appropriate follow-up schedule and administer treatment in a timely manner to maximize the treatment benefits and prevent permanent visual impairment. Besides, it may potentially reduce unnecessary outpatient follow-ups and properly manage medical resources, thereby alleviating the workload of doctors and improving clinical efficiency.

Our study describes a novel application of machine learning and big data mining that benefits patients at risk in clinical practice. Recurrent CSC is often associated with diffuse pathological changes in the RPE, leading to secondary subretinal neovascularization and even permanent vision loss (Wang et al., 2008). Several risk factors for CSC recurrence have been identified, such as male sex, age, sleep disorders, and Type-A behavior (Yannuzzi, 1987; Wang et al., 2008). However, none of these previous studies showed us when the disease would recur; predicting recurrence at a certain time during the course of CSC is still an unsolved and very challenging problem, even for an experienced ophthalmologist. In our study, machine learning helps define the time-related factors regarding CSC recurrence and helps ophthalmologists obtain a more comprehensive understanding of the disease. It is the retinochoroid characteristics [e.g., ChT (choroid thickness), CMT (central macular thickness), and SRF] that have a greater impact on the 3-month short-term recurrence, whereas the life factors (e.g., Pittsburgh Sleep Quality Index and Hamilton Anxiety Scale Score) have a greater impact on the 6-month long-term recurrence (Supplementary Figures 1–10). Machine learning algorithms based on big data are likely to provide us with new strategies that differ from those of clinical research.

Most risk factors identified in previous studies were inferred from analyses of short-term longitudinal data or comparisons of control and intervention groups in randomized trials with a limited number of cases (Otsuka et al., 2002; van Rijssen et al., 2019; Yu et al., 2019). Therefore, the available data are generalizable among only patients with CSC who meet the inclusion and exclusion criteria of the specific studies (Wang et al., 2008). In our study, the inclusion criteria were relatively broad, and we did not include restrictions regarding visual acuity (VA), the duration of the disease, or the number or type of previous treatments. Consequently, the model can be applied to a wide range of patients and scenarios.

Appropriate feature selection is crucial for machine learning models to achieve high accuracy. The recurrence of CSC was predicted with high accuracy after a precise analysis of 165 influencing factors, which also helped us to accurately and comprehensively identify the risk factors for recurrence (Supplementary Figures 1–10). The most significant predictor of CSC recurrence was the Pittsburgh Sleep Quality Index score, and characteristics regarding the duration of CSC and existence of DLS at baseline were also critical for the prediction algorithms, which is consistent with the findings of previous studies (Otsuka et al., 2002; Wang et al., 2008; Yu et al., 2019). In contrast, the characteristics of the angiography data (FFA and ICGA) were less important, and referrals for angiography are often avoided due to the invasiveness of the test (Wang et al., 2008; van Rijssen et al., 2019). Therefore, we removed the features extracted from the angiographic images when we trained the simplified prediction model. OCTA data were also excluded because the examination equipment was not widely available. Therefore, only OCT images and limited clinical data were applied to predict recurrence in a patient with CSC. The simplified prediction model would extend the applications of our model to hospitals in underdeveloped areas without access to FFA, ICGA, or OCTA.

In addition to selective feature extraction, feature weight averaging is also important to minimize instability in classification tasks. We integrated the strengths of the three best-performing original algorithms to obtain a new blended algorithm, which not only improves the accuracy of the blended algorithm but also enhances the stability of the original algorithms. Although the ensemble algorithm performs the best, all the individual algorithms in the full and simplified models achieve high accuracy, demonstrating the stability of our results, the feasibility of our approach, and the exactitude of feature selection.

The limitations of our research should be considered. To further improve predictive accuracy, studies with a longer follow-up and a larger number of CSC patients need to be conducted to augment the dataset for training and validation. Additionally, more studies are warranted to ensure the accuracy of the prediction models in real-world settings.

In summary, our study showed that multidimensional patterns of clinical data and imaging features are predictive factors for CSC recurrence. Our work demonstrates a novel application of machine learning for identifying disease progression and managing follow-ups, thereby preventing vision loss caused by delayed detections and interventions.

## Data Availability Statement

The raw data supporting the conclusions of this article will be made available by the authors, without undue reservation.

## Author Contributions

FX, CW, LZ, CJ, and HL: conception and design. HL, CJ, and DT: administrative support. CJ, FX, and HL: provision of study materials or patients. FX, CJ, YX, LZhou, ZL, SG, CL, LZhang, LL, YG, and XZ: collection and assembly of data. CW, QY, CG, LZhao, FX, YZ, CC, KL, CH, and HZ: data analysis and interpretation. All authors: manuscript writing and final approval of the manuscript.

## Conflict of Interest

The authors declare that the research was conducted in the absence of any commercial or financial relationships that could be construed as a potential conflict of interest.

## Publisher’s Note

All claims expressed in this article are solely those of the authors and do not necessarily represent those of their affiliated organizations, or those of the publisher, the editors and the reviewers. Any product that may be evaluated in this article, or claim that may be made by its manufacturer, is not guaranteed or endorsed by the publisher.
