# Supplementary material for: Predicting Central Serous Chorioretinopathy Recurrence Using Machine Learning
Source: Front Physiol. 2021 Nov 25;12:649316. doi: 10.3389/fphys.2021.649316 (PMC8656454; doi:10.3389/fphys.2021.649316)
Supplement: Supplementary file 1 [file Data_Sheet_1.DOCX]

Supplemental Materials

[Six separated regression algorithms 2](#_Toc30900043)

[Decision Tree 2](#_Toc30900044)

[AdaBoost.R2 2](#_Toc30900045)

[Gradient Boosting 2](#_Toc30900046)

[XGBoost 3](#_Toc30900047)

[Random Forest 3](#_Toc30900048)

[Extra-Trees 3](#_Toc30900049)

[Parameters of each algorithm 4](#_Toc30900043)

[Table S1 5](#_Toc30900050)

[Table S2 8](#_Toc30900051)

[Figure S1 10](#_Toc30900052)

[Figure S2 11](#_Toc30900053)

[Figure S3 12](#_Toc30900054)

[Figure S4 13](#_Toc30900055)

[Figure S5 14](#_Toc30900056)

[Figure S6 15](#_Toc30900057)

[Figure S7 16](#_Toc30900056)

[Figure S8 17](#_Toc30900056)

[Figure S9 18](#_Toc30900056)

[Figure S10 19](#_Toc30900056)

References [20](#_Toc30900056)

**The following six separate regression algorithms were used for recurrence predictions:**

Decision Tree:^1^ Decision tree is a basic classification method with a tree structure. Classification problems can be regarded as sets of if-then rules. In every decision tree, all instances are covered by a path or rules. Generally, decision tree learning includes three steps: feature selection, decision tree generation, and decision tree pruning.

AdaBoost.R2:^2^ AdaBoost is a boosting algorithm based on the idea of fitting a sequence of weak learners by iterating over the same training set, and the final prediction results are obtained by calculating the weighted combination of the outputs of these weak learners. In the implementation of boosting algorithms, the weight of a sample with poor performance in the previous learner is increased, and the updated sample is then used to retrain the next weak learner. When combining all learners, the weight of each weak learner is decided based on its performance.

Gradient Boosting:^3^ Gradient Boosting is a generalization of boosting to arbitrary differentiable loss functions. In this method, the negative gradient of the loss function (the first derivative of the loss function) is used as a measure of the performance of a weak learner, and the weak learner is optimized by reducing the loss function in the direction of the gradient.

Extreme Gradient Boosting:^4^ Extreme Gradient Boosting (XGBoost) is an optimized distributed gradient boosting algorithm implemented based on the original Gradient Boosting framework. Instead of the first derivative, which is used in Gradient Boosting, the first and second-order Taylor expansions of the loss function are used in the optimization process in XGBoost. Consequently, its accuracy is higher, and fewer iterations are required to achieve satisfactory results. Unlike other boosting methods, XGBoost is able to use multithreading when choosing the best segmentation point. The parallel tree boosting operation substantially reduces the run time.

Random Forest:^5^ The Random Forest algorithm is a variant of the bagging (Bootstrap AGGregatING) algorithm that obtains its final results by averaging the predictions of many decision trees. The training set used to construct each decision tree is obtained using the bootstrap method (random sampling with replacement from the original data). Furthermore, when splitting one node during the construction of a tree, a subset of all features at that node is randomly selected, and then an optimal feature is selected from this subset for splitting. Because of the use of random sampling and random feature selection, the Random Forest algorithm is not easily susceptible to overfitting, although no pruning is performed on any single tree.

Extra-Trees:^6^ The Extremely Randomized Trees (Extra-Trees) algorithm is a variant of the Random Forest algorithm obtained by introducing random thresholds when splitting nodes. The Random Forest algorithm uses the bootstrap method to obtain the training set, whereas Extra-Trees uses all samples for training. Instead of choosing the most discriminative thresholds in feature subsets, as in the Random Forest algorithm, Extra-Trees randomly selects thresholds for the candidate features and then selects the best threshold for node splitting.

**Some of the parameters considered in this study for each algorithm are listed below:**

AdaBoost.R2: learning_rate=0.5, n_estimators=50, max_depth=3; Gradient Boosting: learning_rate=0.1, n_estimators=50, max_depth=3; XGBoost: learning_rate=0.1, n_estimators=500, max_depth=9; Random Forest: n_estimators=500, max_depth=80, max_features='sqrt'; and Extra-Trees: same parameters as the Random Forest algorithm. Here, learning_rate represents the learning rate of the algorithm, n_estimators represents the number of weak learners (decision trees), max_depth represents the maximum depth of a decision tree, and max_features denotes the size of the random subsets used for node splitting. We used a random seed (seed=0) for each algorithm to ensure the reproducibility of the predictions.

**Table S1. Clinical Records and Imaging Features Used to Predict Recurrence**

| **Clinical Data** | | | | **Features from FFA and ICGA** | | **Features from OCTA** | |
| --- | --- | --- | --- | --- | --- | --- | --- |
| **Feature** | **Description** | **Feature** | **Description** | **Feature** | **Description** | **Feature** | **Description** |
| Age  Sex  Height  Weight  Education  Income  Heart Disease  Gastropathy  Autoimmune Disease  Steroid Usage  Hamilton Anxiety Scale  Pittsburgh Sleep Quality Index | Age of the patient  Sex of the patient  Height of the patient  Weight of the patient  Education level of the patient  Income level of the patient  History of heart disease  History of gastropathy  History of autoimmune disease  History of steroid use  Hamilton Anxiety Scale^7^ score  Pittsburgh Sleep Quality Index^8^ score | Type-A Behavior  Eye  Duration  Therapy  VA Baseline  VA 1-mo  VA 3-mo  VA 6-mo | Type-A Behavior^9^ score  Right or left eye  Duration of CSC  hd-PDT, SML or CL  VA before treatment  VA at 1-mo after treatment  VA at 3-mo after treatment  VA at 6-mo after treatment | FFA leakage  Single or multiple  Morphology  Area  Position 1  ICGA leakage  High permeability  Position 2  Low permeability  Position 3 | Existence of active leakage at baseline  No. of active leakage sites on FFA  Morphology of the leakage on FFA  Area of the leakage on FFA  Position of the leakage on FFA  Existence of active leakage at baseline  High permeability on ICGA  Position of the high permeability on ICGA  Low permeability on ICGA  Position of the low permeability on ICGA | High reflection  Position 4  Low reflection  Position 5  BVN Baseline  Position 6  BVN 1-mo  Position 7  BVN 3-mo  Position 8  BVN 6-mo  Position 9 | Existence of high reflection at baseline  Position of high reflection on OCTA  Existence of low reflection at baseline  Position of low reflection on OCTA  Existence of BVN at baseline  Position of BVN at baseline  Existence of BVN at 1-mo  Position of BVN at 1-mo  Existence of BVN at 3-mo  Position of BVN at 3-mo  Existence of BVN at 6-mo  Position of BVN at 6-mo |
| **Features from OCT (Baseline)** | | **Features from OCT (1-mo)** | | **Features from OCT (3-mo)** | | **Features from OCT (6-mo)** | |
| **Feature** | **Description** | **Feature** | **Description** | **Feature** | **Description** | **Feature** | **Description** |
| SFA horizontal  SFA vertical  SFA  CMT horizontal  CMT vertical  CMT  RNEL horizontal  RNEL vertical  RNEL  SRF horizontal  SRF vertical  SRF  ChT horizontal  ChT vertical  ChT  EZ horizontal  EZ vertical  EZ  PED horizontal  PED vertical  PED  DLS horizontal  DLS vertical  DLS  Bruch’s membrane horizontal  Bruch’s membrane vertical  Bruch’s membrane | Subretinal fluid absorption on horizontal B-scan  Subretinal fluid absorption on vertical B-scan  Subretinal fluid absorption at baseline  CMT on horizontal B-scan  CMT on vertical B-scan  Average CMT at baseline  Thickness of RNEL on horizontal B-scan  Thickness of RNEL on vertical B-scan  Average thickness of RNEL at baseline  Height of SRF on horizontal B-scan  Height of SRF on vertical B-scan  Average height of SRF at baseline  ChT on horizontal B-scan  ChT on vertical B-scan  Average ChT at baseline  Integrity of EZ on horizontal B-scan  Integrity of EZ on vertical B-scan  Average integrity of EZ at baseline  Existence of PED on horizontal B-scan  Existence of PED on vertical B-scan  Existence of PED at baseline  Existence of DLS on horizontal B-scan  Existence of DLS on vertical B-scan  Existence of DLS at baseline  Bruch’s membrane on horizontal B-scan  Bruch’s membrane on vertical B-scan  Bruch’s membrane at baseline | SFA horizontal  SFA vertical  SFA  CMT horizontal  CMT vertical  CMT  RNEL horizontal  RNEL vertical  RNEL  SRF horizontal  SRF vertical  SRF  ChT horizontal  ChT vertical  ChT  ChT (1-mo - B) horizontal  ChT (1-mo - B) vertical  ChT (1-mo - B)  EZ horizontal  EZ vertical  EZ  PED horizontal  PED vertical  PED  DLS horizontal  DLS vertical  DLS  Bruch’s membrane horizontal  Bruch’s membrane vertical  Bruch’s membrane | Subretinal fluid absorption on horizontal B-scan  Subretinal fluid absorption on vertical B-scan  Subretinal fluid absorption at 1-mo  CMT on horizontal B-scan  CMT on vertical B-scan  Average CMT at 1-mo  Thickness of RNEL on horizontal B-scan  Thickness of RNEL on vertical B-scan  Average thickness of RNEL at 1-mo  Height of SRF on horizontal B-scan  Height of SRF on vertical B-scan  Average height of SRF at 1-mo  ChT on horizontal B-scan  ChT on vertical B-scan  Average ChT at 1-mo  ChT variation (1-mo - baseline) on horizontal B-scan  ChT variation (1-mo - baseline) on vertical B-scan  Average ChT variation (1-mo - baseline) at 1-mo  Integrity of EZ on horizontal B-scan  Integrity of EZ on vertical B-scan  Average integrity of EZ at 1-mo  Existence of PED on horizontal B-scan  Existence of PED on vertical B-scan  Existence of PED at 1-mo  Existence of DLS on horizontal B-scan  Existence of DLS on vertical B-scan  Existence of DLS at 1-mo  Bruch’s membrane on horizontal B-scan  Bruch’s membrane on vertical B-scan  Bruch’s membrane at 1-mo | SFA horizontal  SFA vertical  SFA  CMT horizontal  CMT vertical  CMT  RNEL horizontal  RNEL vertical  RNEL  SRF horizontal  SRF vertical  SRF  ChT horizontal  ChT vertical  ChT  ChT (3-mo – 1-mo) horizontal  ChT (3-mo – 1-mo) vertical  ChT (3-mo – 1-mo)  EZ horizontal  EZ vertical  EZ  PED horizontal  PED vertical  PED  DLS horizontal  DLS vertical  DLS  Bruch’s membrane horizontal  Bruch’s membrane vertical  Bruch’s membrane  Recurrence horizontal  Recurrence vertical  Recurrence | Subretinal fluid absorption on horizontal B-scan  Subretinal fluid absorption on vertical B-scan  Subretinal fluid absorption at 3-mo  CMT on horizontal B-scan  CMT on vertical B-scan  Average CMT at 3-mo  Thickness of RNEL on horizontal B-scan  Thickness of RNEL on vertical B-scan  Average thickness of RNEL at 3-mo  Height of SRF on horizontal B-scan  Height of SRF on vertical B-scan  Average height of SRF at 3-mo  ChT on horizontal B-scan  ChT on vertical B-scan  Average ChT at 3-mo  ChT variation (3-mo – 1-mo) on horizontal B-scan  ChT variation (3-mo – 1-mo) on vertical B-scan  Average ChT variation (3-mo – 1-mo) at 3-mo  Integrity of EZ on horizontal B-scan  Integrity of EZ on vertical B-scan  Average integrity of EZ at 3-mo  Existence of PED on horizontal B-scan  Existence of PED on vertical B-scan  Existence of PED at 3-mo  Existence of DLS on horizontal B-scan  Existence of DLS on vertical B-scan  Existence of DLS at 3-mo  Bruch’s membrane on horizontal B-scan  Bruch’s membrane on vertical B-scan  Bruch’s membrane at 3-mo  Recurrence on horizontal B-scan  Recurrence on vertical B-scan  Recurrence at 3-mo | SFA horizontal  SFA vertical  SFA  CMT horizontal  CMT vertical  CMT  RNEL horizontal  RNEL vertical  RNEL  SRF horizontal  SRF vertical  SRF  ChT horizontal  ChT vertical  ChT  ChT (6-mo – 3-mo) horizontal  ChT (6-mo – 3-mo) vertical  ChT (6-mo – 3-mo)  EZ horizontal  EZ vertical  EZ  PED horizontal  PED vertical  PED  DLS horizontal  DLS vertical  DLS  Bruch’s membrane horizontal  Bruch’s membrane vertical  Bruch’s membrane  Recurrence horizontal  Recurrence vertical  Recurrence | Subretinal fluid absorption on horizontal B-scan  Subretinal fluid absorption on vertical B-scan  Subretinal fluid absorption at 6-mo  CMT on horizontal B-scan  CMT on vertical B-scan  Average CMT at 6-mo  Thickness of RNEL on horizontal B-scan  Thickness of RNEL on vertical B-scan  Average thickness of RNEL at 6-mo  Height of SRF on horizontal B-scan  Height of SRF on vertical B-scan  Average height of SRF at 6-mo  ChT on horizontal B-scan  ChT on vertical B-scan  Average ChT at 6-mo  ChT variation (6-mo – 3-mo) on horizontal B-scan  ChT variation (6-mo – 3-mo) on vertical B-scan  Average ChT variation (6-mo – 3-mo) at 6-mo  Integrity of EZ on horizontal B-scan  Integrity of EZ on vertical B-scan  Average integrity of EZ at 6-mo  Existence of PED on horizontal B-scan  Existence of PED on vertical B-scan  Existence of PED at 6-mo  Existence of DLS on horizontal B-scan  Existence of DLS on vertical B-scan  Existence of DLS at 6-mo  Bruch’s membrane on horizontal B-scan  Bruch’s membrane on vertical B-scan  Bruch’s membrane at 6-mo  Recurrence on horizontal B-scan  Recurrence on vertical B-scan  Recurrence at 6-mo |

This table shows all 20 clinical records and 145 imaging features used to train and validate the models. Twenty records (e.g., duration) were retrieved from the electronic medical records, 5 features (e.g., position and area of the leakage point) were calculated from FFA (early, middle and late phases), 5 features (e.g., hyperperfusion and hypoperfusion) were calculated from ICGA (early, middle and late phases), 12 features (e.g., the existence of abnormal reflection and BVN) were calculated from OCTA (superficial choroidal layer) and 123 features (e.g., DLS, CMT, EZ and ChT) were calculated from OCT. Please see Figure 1 for a visual representation of the measured features. VA, visual acuity; OCT, optical coherence tomography; OCTA, optical coherence tomography angiography; CSC, central serous chorioretinopathy; CL, conventional laser; SML, subthreshold micropulse laser; hd-PDT, half-dose photodynamic therapy; FFA, fundus fluorescein angiography; Single or multiple, a label of 1 indicates the existence of a single leakage point, and 2 indicates multiple leakage sites; Morphology, a label of 1 indicates smokestack leakage on FFA, 2 indicates focal diffuse leakage, and 3 indicates multiple diffuse leakage sites; Area, a label of 1 indicates that the area of leakage on FFA was smaller than the area of the optic disc, and 2 indicates a larger area; Position (position 1 to position 9), a label of 1 indicates that the damage was located less than 1500 microns away from the fovea and 2 indicates a distance greater than 1500 microns; ICGA, indocyanine green angiography; High permeability, a label of 1 indicates the existence of high permeability, and 2 indicates normal permeability; Low permeability, a label of 1 indicates the existence of low permeability, and 2 indicates normal permeability; High reflection, a label of 1 indicates the existence of high reflection on OCTA, and 2 indicates normal reflection; Low reflection, a label of 1 indicates the existence of low reflection on OCTA, and 2 indicates normal reflection; BVN, branching vascular network, a label of 1 indicates the existence of BVN, and 2 indicates a normal structure. All OCTA features are derived from images of the superficial choroidal layer, which is defined as 10 microns above Bruch’s membrane to 30 microns below Bruch’s membrane in the 3*3 scanning pattern of Optovue (version 2017.1.0.155) software. SRF, subretinal fluid; CMT, central macular thickness; RNEL, retinal neuroepithelial layer; ChT, choroidal thickness, all measurements are expressed in microns; SFA, subretinal fluid absorption, a label of 1 indicates an increase in the level of unabsorbed SRF, 2 indicates partially absorbed SRF, and 3 indicates completely absorbed SRF; EZ, ellipsoid zone, a label of 1 indicates the complete absence of the original neurosensory retinal detachment area, 2 indicates the intermittent existence of the original neurosensory retinal detachment area with less than half of the total length, 3 indicates the existence of most of the original neurosensory retinal detachment area, and 4 indicates the complete existence of the original neurosensory retinal detachment area; PED, retinal pigment epithelial detachment, a label of 1 indicates the existence of PED, and 2 indicates a normal structure; DLS, double-layer sign, a label of 1 indicates the existence of DLS, and 2 indicates a normal structure; Bruch's membrane, a label of 1 indicates disruption of Bruch's membrane, and 2 indicates a normal membrane; Recurrence, a label of 1 indicates the reappearance of SRF, and 2 indicates a normal structure on OCT (in the analysis of quantitative data, we used the mean values of horizontal and vertical B-scans on OCT; in the analysis of qualitative data, we used the worse values of the horizontal and vertical B-scans on OCT).

| **Clinical Data** | | | | | | | |
| --- | --- | --- | --- | --- | --- | --- | --- |
| **Feature** | **Description** | **Feature** | **Description** | **Feature** | **Description** | **Feature** | **Description** |
| Age  Education  Income | Age of the patient  Education level of the patient  Income level of the patient | Hamilton Anxiety Scale  Pittsburgh Sleep Quality Index  Duration | Hamilton Anxiety Scale^7^ score  Pittsburgh Sleep Quality Index^8^ score  Duration of CSC | Therapy  VA Baseline  VA 1-mo | CL, SML, or hd-PDT  VA before treatment  VA at 1-mo after treatment | VA 3-mo  VA 6-mo | VA at 3-mo after treatment  VA at 6-mo after treatment |
| **Features from OCT (Baseline)** | | **Features from OCT (1-mo)** | | **Features from OCT (3-mo)** | | **Features from OCT (6-mo)** | |
| **Feature** | **Description** | **Feature** | **Description** | **Feature** | **Description** | **Feature** | **Description** |
| SFA horizontal  SFA vertical  SFA  CMT horizontal  CMT vertical  CMT  RNEL horizontal  RNEL vertical  RNEL  SRF horizontal  SRF vertical  SRF  ChT horizontal  ChT vertical  ChT  EZ horizontal  EZ vertical  EZ  PED horizontal  PED vertical  PED  DLS horizontal  DLS vertical  DLS  Bruch’s membrane horizontal  Bruch’s membrane vertical  Bruch’s membrane | Subretinal fluid absorption on horizontal B-scan  Subretinal fluid absorption on vertical B-scan  Subretinal fluid absorption at baseline  CMT on horizontal B-scan  CMT on vertical B-scan  Average CMT at baseline  Thickness of RNEL on horizontal B-scan  Thickness of RNEL on vertical B-scan  Average thickness of RNEL at baseline  Height of SRF on horizontal B-scan  Height of SRF on vertical B-scan  Average height of SRF at baseline  ChT on horizontal B-scan  ChT on vertical B-scan  Average ChT at baseline  Integrity of EZ on horizontal B-scan  Integrity of EZ on vertical B-scan  Average integrity of EZ at baseline  Existence of PED on horizontal B-scan  Existence of PED on vertical B-scan  Existence of PED at baseline  Existence of DLS on horizontal B-scan  Existence of DLS on vertical B-scan  Existence of DLS at baseline  Bruch’s membrane on horizontal B-scan  Bruch’s membrane on vertical B-scan  Bruch’s membrane at baseline | SFA horizontal  SFA vertical  SFA  CMT horizontal  CMT vertical  CMT  RNEL horizontal  RNEL vertical  RNEL  SRF horizontal  SRF vertical  SRF  ChT horizontal  ChT vertical  ChT  ChT (1-mo - B) horizontal  ChT (1-mo - B) vertical  ChT (1-mo - B)  EZ horizontal  EZ vertical  EZ  PED horizontal  PED vertical  PED  DLS horizontal  DLS vertical  DLS  Bruch’s membrane horizontal  Bruch’s membrane vertical  Bruch’s membrane | Subretinal fluid absorption on horizontal B-scan  Subretinal fluid absorption on vertical B-scan  Subretinal fluid absorption at 1-mo  CMT on horizontal B-scan  CMT on vertical B-scan  Average CMT at 1-mo  Thickness of RNEL on horizontal B-scan  Thickness of RNEL on vertical B-scan  Average thickness of RNEL at 1-mo  Height of SRF on horizontal B-scan  Height of SRF on vertical B-scan  Average height of SRF at 1-mo  ChT on horizontal B-scan  ChT on vertical B-scan  Average ChT at 1-mo  ChT variation (1-mo - baseline) on horizontal B-scan  ChT variation (1-mo - baseline) on vertical B-scan  Average ChT variation (1-mo - baseline) at 1-mo  Integrity of EZ on horizontal B-scan  Integrity of EZ on vertical B-scan  Average integrity of EZ at 1-mo  Existence of PED on horizontal B-scan  Existence of PED on vertical B-scan  Existence of PED at 1-mo  Existence of DLS on horizontal B-scan  Existence of DLS on vertical B-scan  Existence of DLS at 1-mo  Bruch’s membrane on horizontal B-scan  Bruch’s membrane on vertical B-scan  Bruch’s membrane at 1-mo | SFA horizontal  SFA vertical  SFA  CMT horizontal  CMT vertical  CMT  RNEL horizontal  RNEL vertical  RNEL  SRF horizontal  SRF vertical  SRF  ChT horizontal  ChT vertical  ChT  ChT (3-mo – 1-mo) horizontal  ChT (3-mo – 1-mo) vertical  ChT (3-mo – 1-mo)  EZ horizontal  EZ vertical  EZ  PED horizontal  PED vertical  PED  DLS horizontal  DLS vertical  DLS  Bruch’s membrane horizontal  Bruch’s membrane vertical  Bruch’s membrane  Recurrence horizontal  Recurrence vertical  Recurrence | Subretinal fluid absorption on horizontal B-scan  Subretinal fluid absorption on vertical B-scan  Subretinal fluid absorption at 3-mo  CMT on horizontal B-scan  CMT on vertical B-scan  Average CMT at 3-mo  Thickness of RNEL on horizontal B-scan  Thickness of RNEL on vertical B-scan  Average thickness of RNEL at 3-mo  Height of SRF on horizontal B-scan  Height of SRF on vertical B-scan  Average height of SRF at 3-mo  ChT on horizontal B-scan  ChT on vertical B-scan  Average ChT at 3-mo  ChT variation (3-mo – 1-mo) on horizontal B-scan  ChT variation (3-mo – 1-mo) on vertical B-scan  Average ChT variation (3-mo – 1-mo) at 3-mo  Integrity of EZ on horizontal B-scan  Integrity of EZ on vertical B-scan  Average integrity of EZ at 3-mo  Existence of PED on horizontal B-scan  Existence of PED on vertical B-scan  Existence of PED at 3-mo  Existence of DLS on horizontal B-scan  Existence of DLS on vertical B-scan  Existence of DLS at 3-mo  Bruch’s membrane on horizontal B-scan  Bruch’s membrane on vertical B-scan  Bruch’s membrane at 3-mo  Recurrence on horizontal B-scan  Recurrence on vertical B-scan  Recurrence at 3-mo | SFA horizontal  SFA vertical  SFA  CMT horizontal  CMT vertical  CMT  RNEL horizontal  RNEL vertical  RNEL  SRF horizontal  SRF vertical  SRF  ChT horizontal  ChT vertical  ChT  ChT (6-mo – 3-mo) horizontal  ChT (6-mo – 3-mo) vertical  ChT (6-mo – 3-mo)  EZ horizontal  EZ vertical  EZ  PED horizontal  PED vertical  PED  DLS horizontal  DLS vertical  DLS  Bruch’s membrane horizontal  Bruch’s membrane vertical  Bruch’s membrane  Recurrence horizontal  Recurrence vertical  Recurrence | Subretinal fluid absorption on horizontal B-scan  Subretinal fluid absorption on vertical B-scan  Subretinal fluid absorption at 6-mo  CMT on horizontal B-scan  CMT on vertical B-scan  Average CMT at 6-mo  Thickness of RNEL on horizontal B-scan  Thickness of RNEL on vertical B-scan  Average thickness of RNEL at 6-mo  Height of SRF on horizontal B-scan  Height of SRF on vertical B-scan  Average height of SRF at 6-mo  ChT on horizontal B-scan  ChT on vertical B-scan  Average ChT at 6-mo  ChT variation (6-mo – 3-mo) on horizontal B-scan  ChT variation (6-mo – 3-mo) on vertical B-scan  Average ChT variation (6-mo – 3-mo) at 6-mo  Integrity of EZ on horizontal B-scan  Integrity of EZ on vertical B-scan  Average integrity of EZ at 6-mo  Existence of PED on horizontal B-scan  Existence of PED on vertical B-scan  Existence of PED at 6-mo  Existence of DLS on horizontal B-scan  Existence of DLS on vertical B-scan  Existence of DLS at 6-mo  Bruch’s membrane on horizontal B-scan  Bruch’s membrane on vertical B-scan  Bruch’s membrane at 6-mo  Recurrence on horizontal B-scan  Recurrence on vertical B-scan  Recurrence at 6-mo |

**Table S2. Clinical Records and Imaging Features Used to Predict Recurrence with the Simplified Model**

This table shows all 11 clinical records and 123 OCT features used to train and validate the models. Eleven features (e.g., duration) were retrieved from electronic records, and 123 features were calculated from OCT. VA, visual acuity; OCT, optical coherence tomography; CSC, central serous chorioretinopathy; CL, conventional laser; SML, subthreshold micropulse laser; hd-PDT, half-dose photodynamic therapy; SRF, subretinal fluid; CMT, central macular thickness; RNEL, retinal neuroepithelial layer; ChT, choroidal thickness, all measurements are expressed in microns; SFA, subretinal fluid absorption, a label of 1 indicates an increase in the level of unabsorbed SRF, 2 indicates partially absorbed SRF, and 3 indicates completely absorbed SRF; EZ, ellipsoid zone, a label of 1 indicates the complete absence of the original neurosensory retinal detachment area, 2 indicates the intermittent existence of the original neurosensory retinal detachment area with less than half of the total length, 3 indicates the existence of most of the original neurosensory retinal detachment area, and 4 indicates complete existence of the original neurosensory retinal detachment area; PED, retinal pigment epithelial detachment, a label of 1 indicates the existence of PED, and 2 indicates a normal structure; DLS, double-layer sign, a label of 1 indicates the existence of DLS, and 2 indicates a normal structure; Bruch's membrane, a label of 1 indicates disruption of Bruch's membrane, and 2 indicates a normal membrane; Recurrence, a label of 1 indicates the reappearance of SRF, and 2 indicates a normal structure on OCT (in the analysis of quantitative data, we used the mean values of horizontal and vertical B-scans on OCT; in the analysis of qualitative data, we used the worse values of the horizontal and vertical B-scans on OCT).

**Figure S1. The Relative Importance of Different Features for Recurrence Prediction with the Full Model**


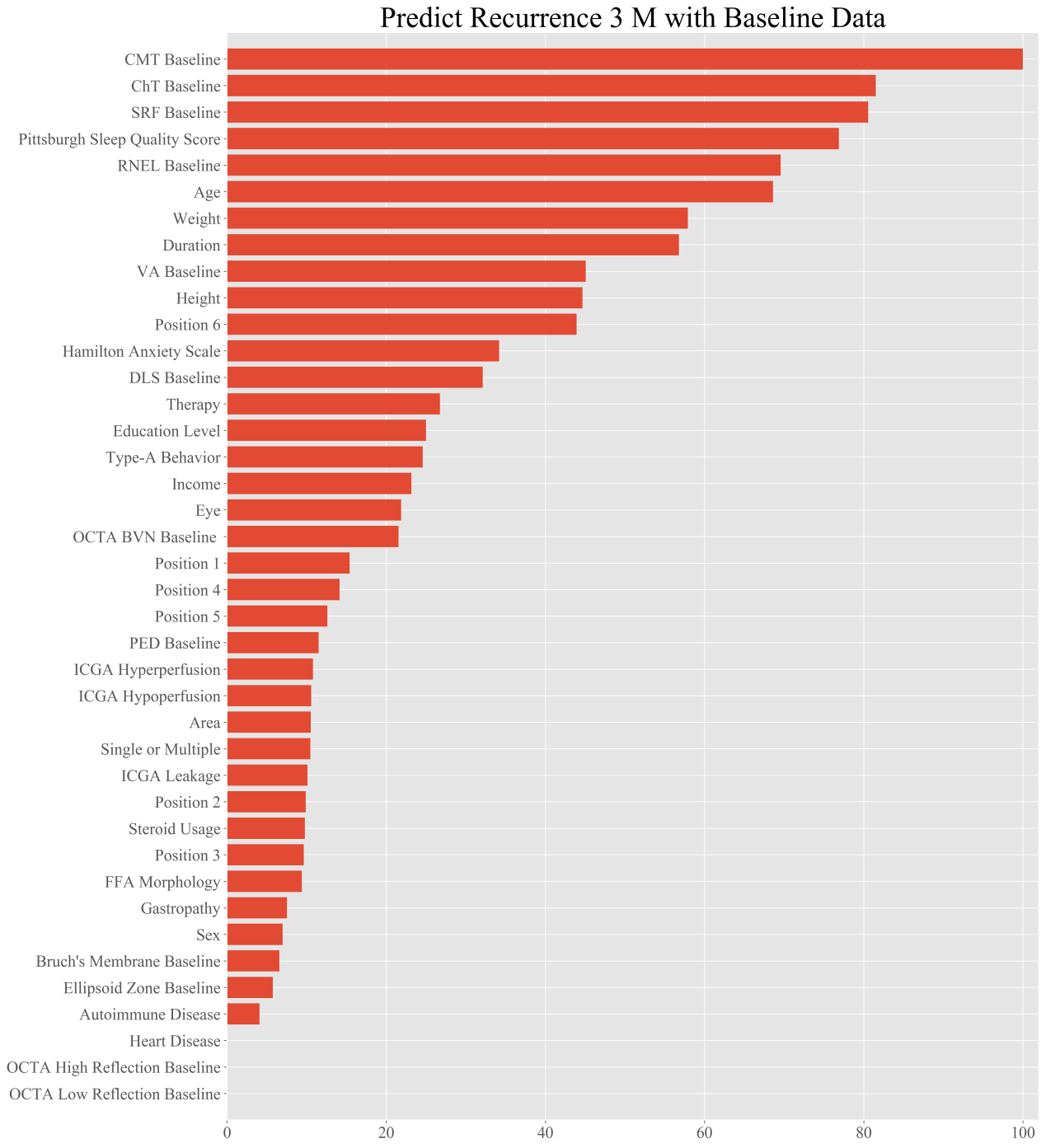


The plot shows the weights of the different features for the recurrence prediction task at 3 months; the red bar indicates the average importance of the feature to the blending algorithm. This figure shows the feature weights in the 3-month recurrence prediction based on the baseline data.

**Figure S2. The Relative Importance of Different Features for Recurrence Prediction with the Full Model**


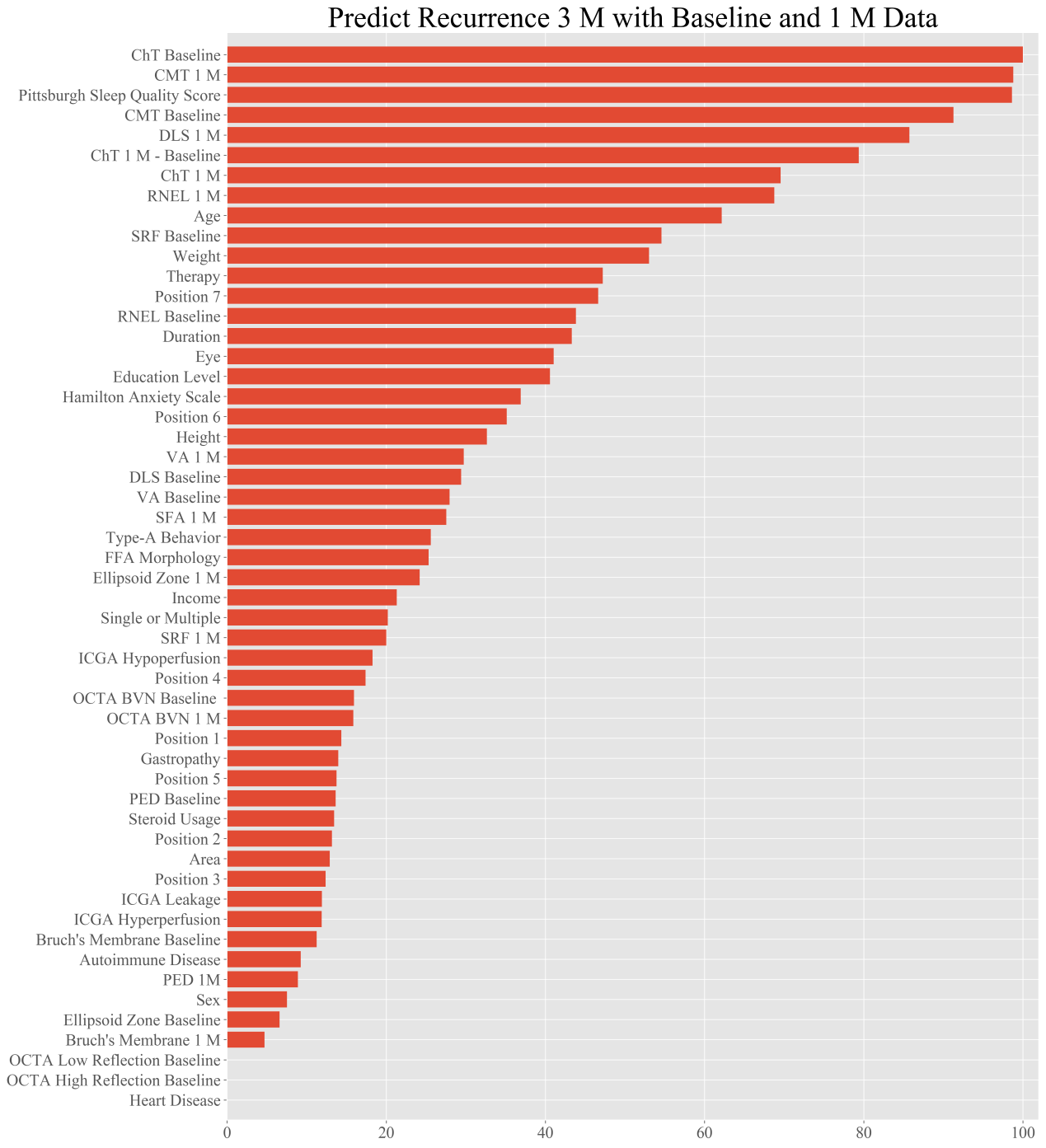


This figure shows the feature weights in the 3-month recurrence prediction based on the data collected at baseline and 1 month.

**Figure S3. The Relative Importance of Different Features for Recurrence Prediction with the Full Model**


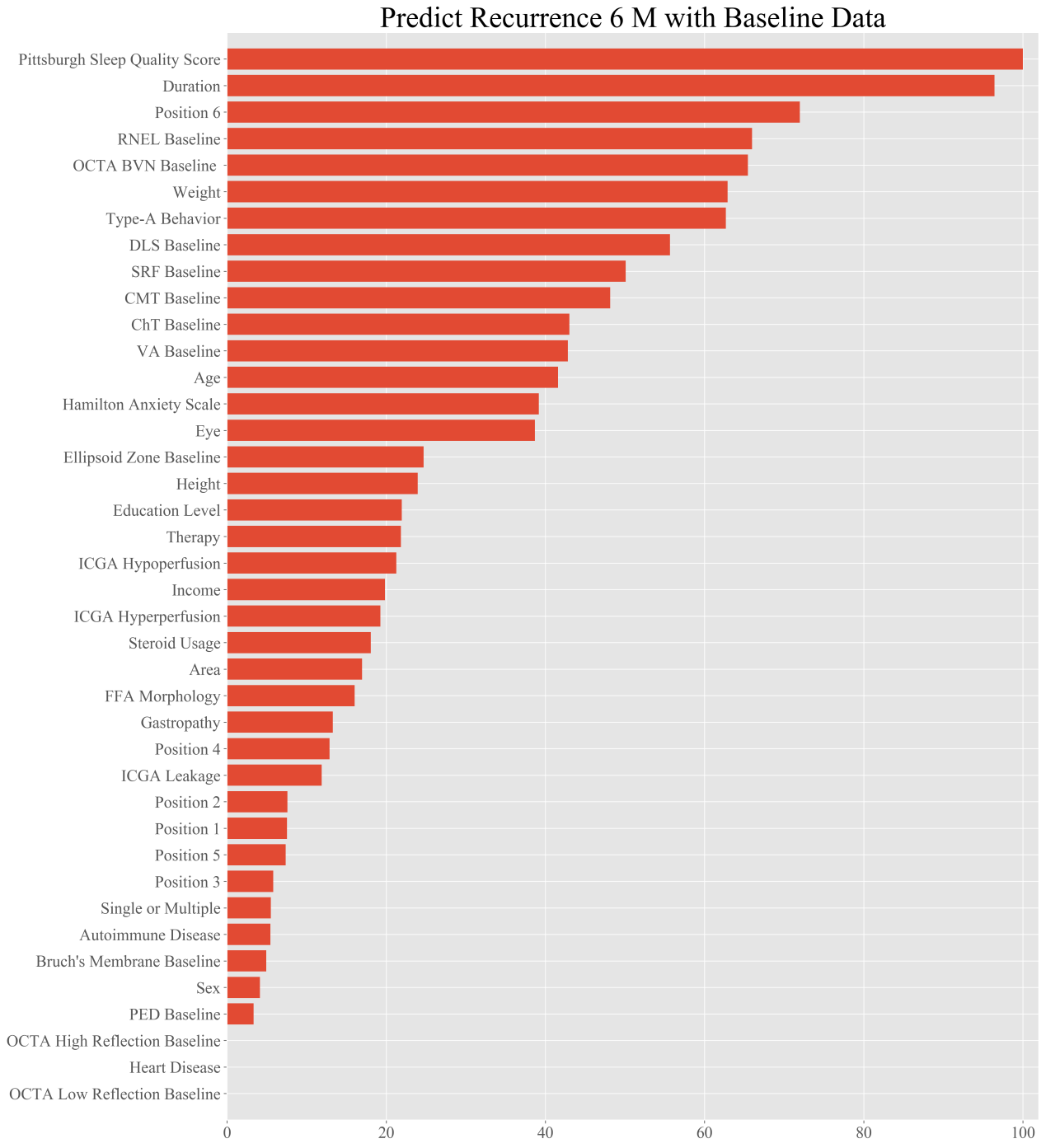


This figure shows the feature weights in the 6-month recurrence prediction based on the baseline data.

**Figure S4. The Relative Importance of Different Features for Recurrence Prediction with the Full Model**


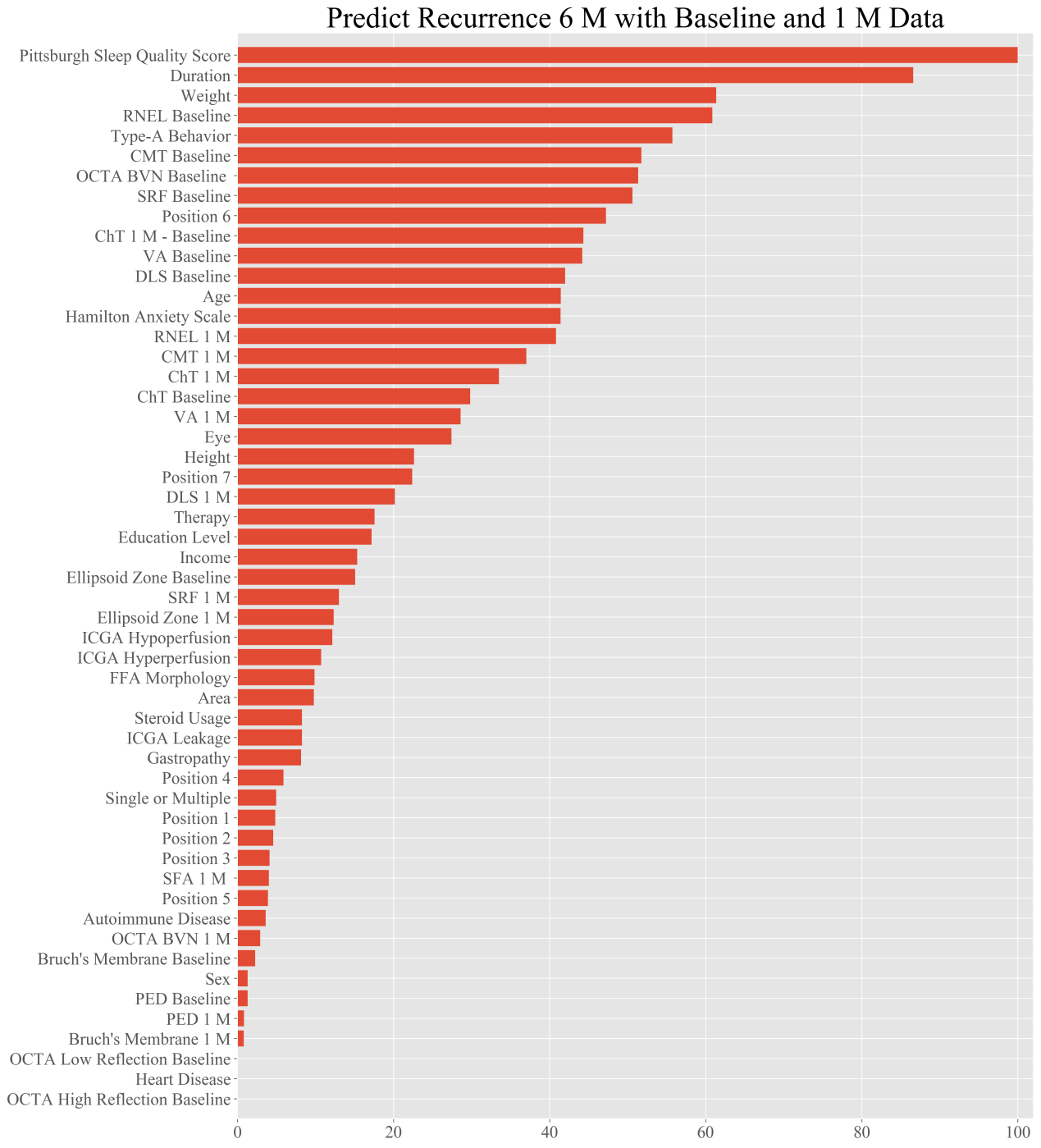


This figure shows the feature weights in the 6-month recurrence prediction based on the data collected at baseline and 1 month.

**Figure S5. The Relative Importance of Different Features for Recurrence Prediction with the Full Model**


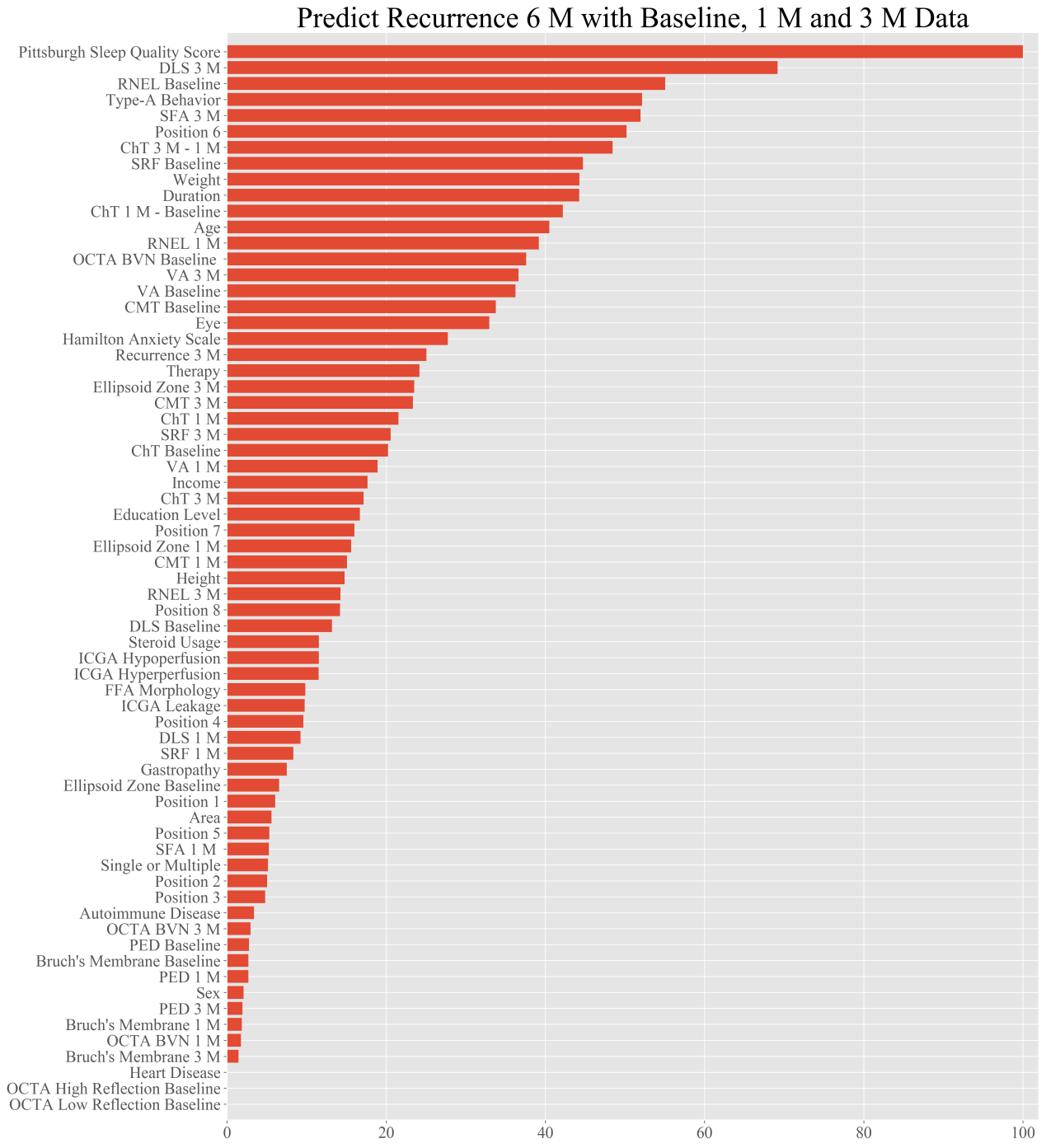


This figure shows the feature weights in the 6-month recurrence prediction based on the data collected at baseline, 1 month and 3 months.

**Figure S6. The Relative Importance of Different Features for Recurrence Prediction with the Simplified Model**


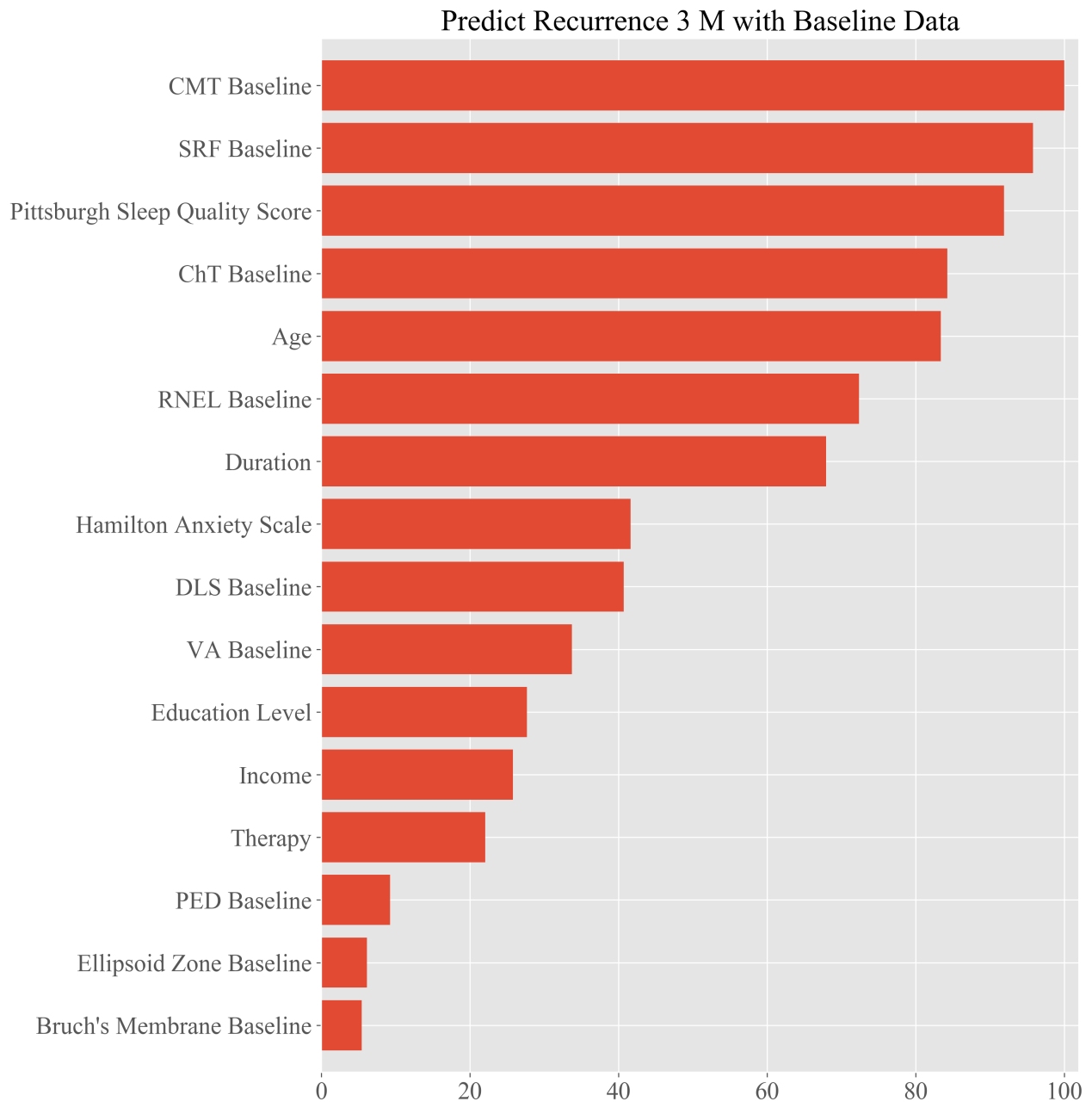


This figure shows the feature weights in the 3-month recurrence prediction based on the baseline data.

**Figure S7. The Relative Importance of Different Features for Recurrence Prediction with the Simplified Model**


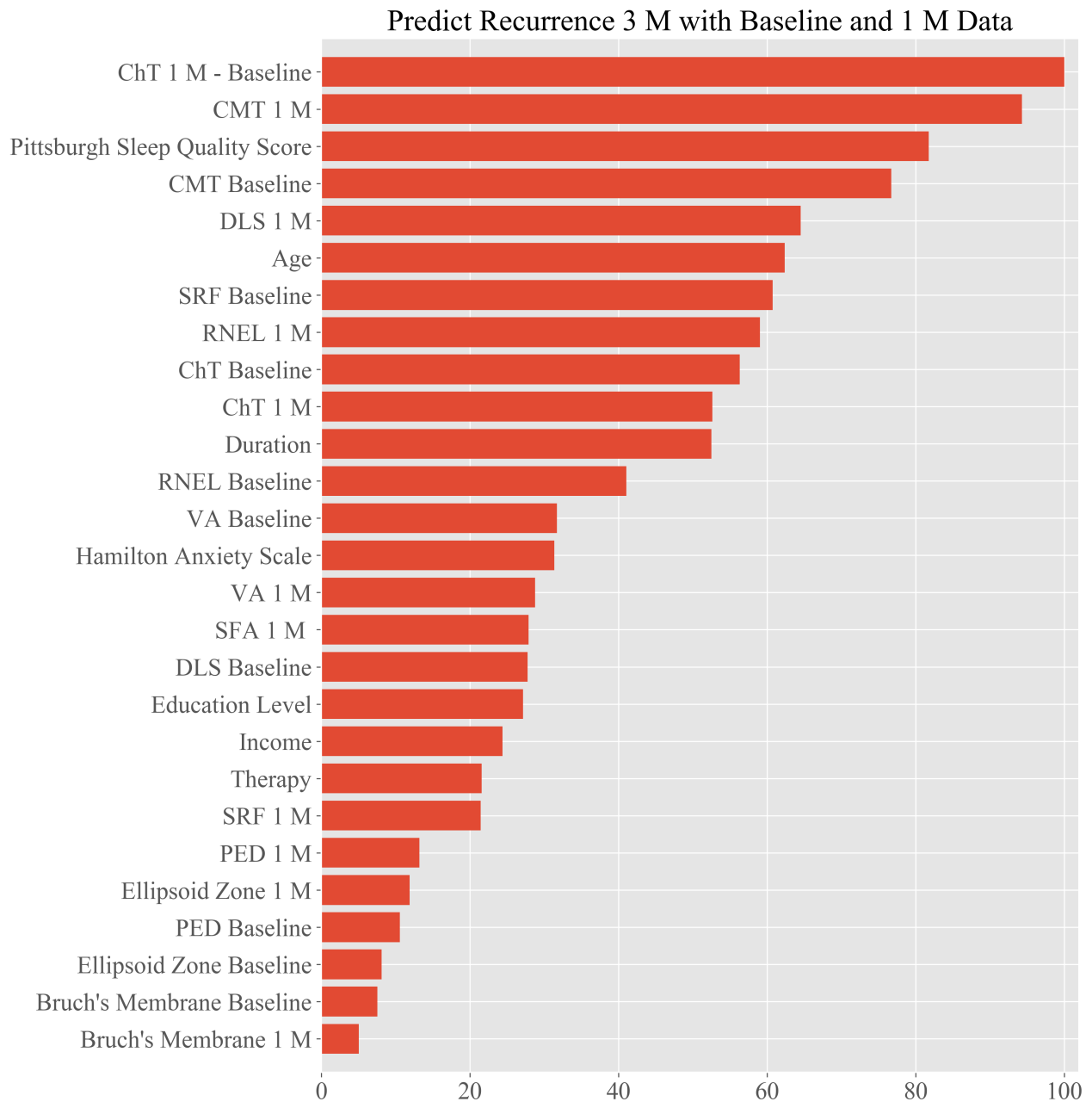


This figure shows the feature weights in the 3-month recurrence prediction based on the data collected at baseline and 1 month.

**Figure S8. The Relative Importance of Different Features for Recurrence Prediction with the Simplified Model**


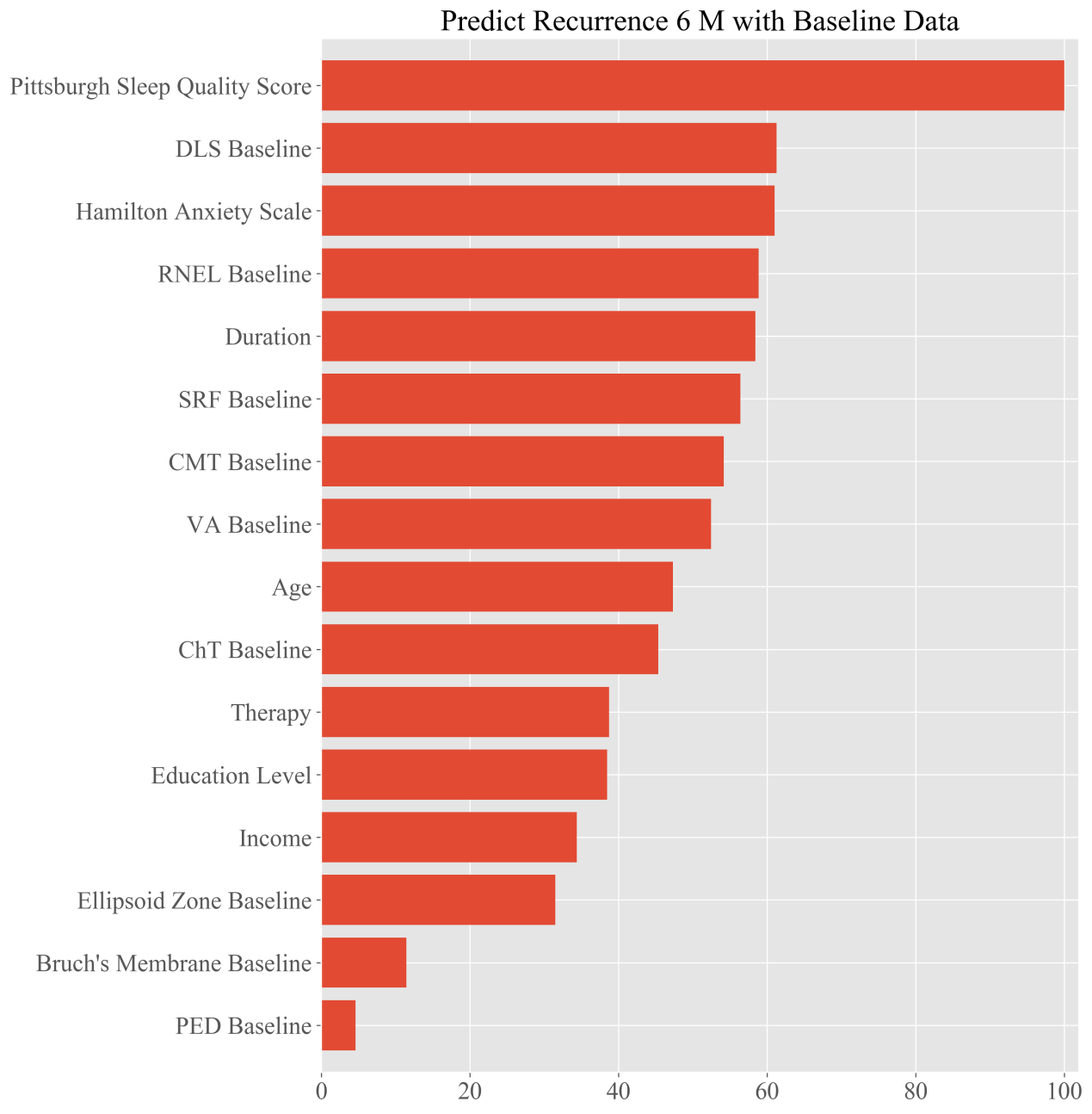


This figure shows the feature weights in the 6-month recurrence prediction based on the baseline data.

**Figure S9. The Relative Importance of Different Features for Recurrence Prediction with the Simplified Model**


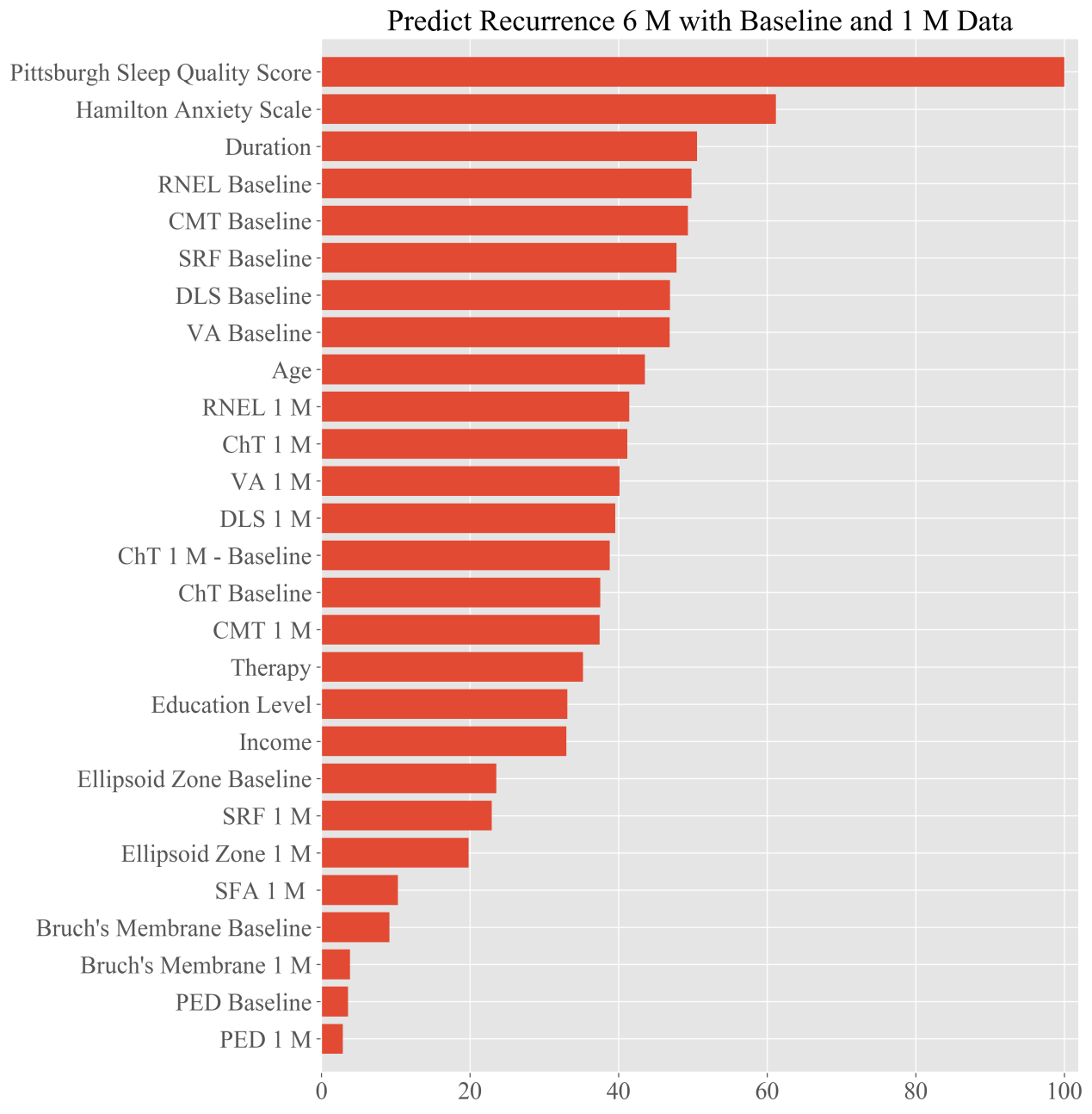


This figure shows the feature weights in the 6-month recurrence prediction based on the data collected at baseline and 1 month.

**Figure S10. The Relative Importance of Different Features for Recurrence Prediction with the Simplified Model**


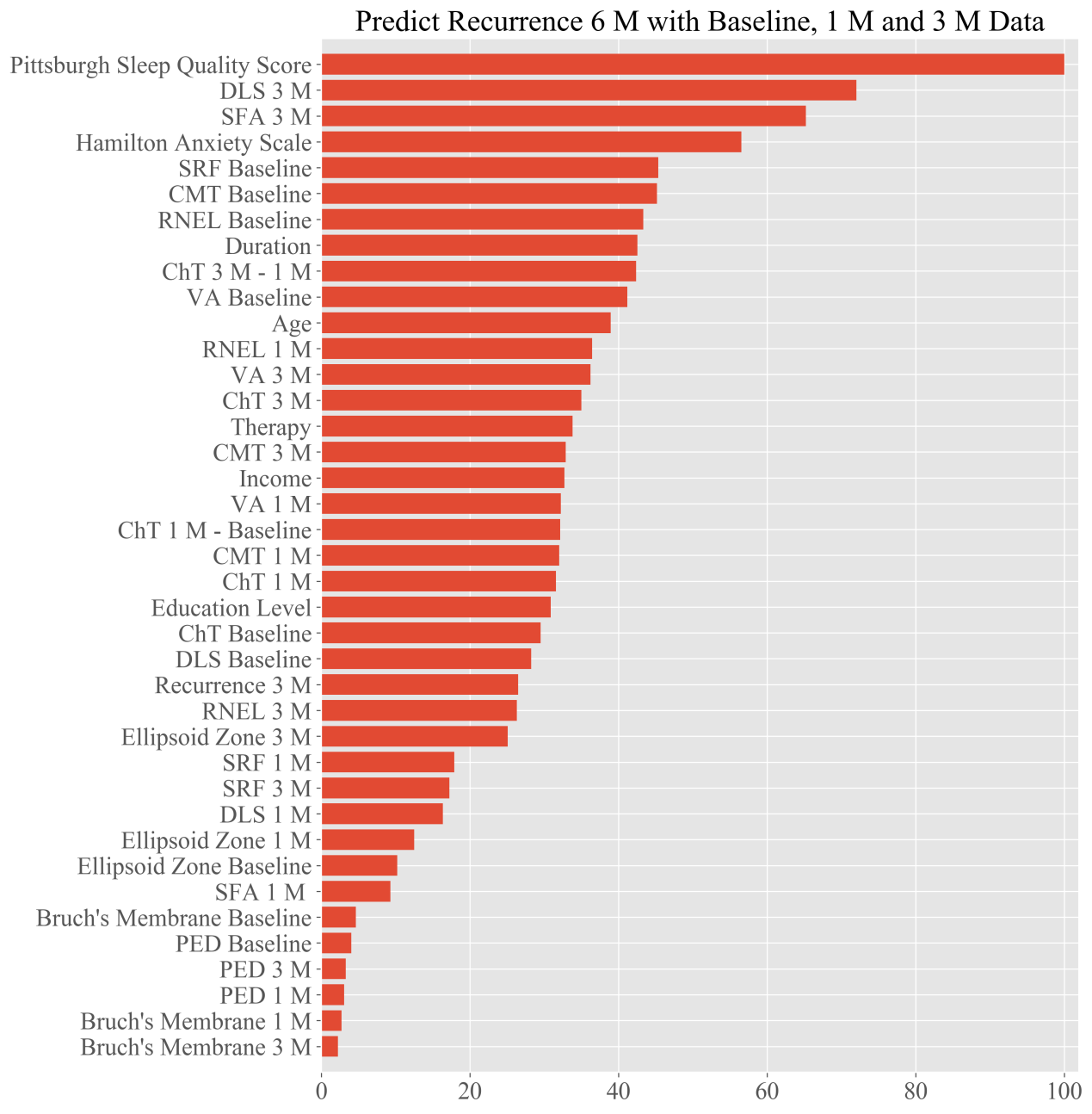


This figure shows the feature weights in the 6-month recurrence prediction based on the data collected at baseline, 1 month and 3 months.

**References**

1. Schoning V, Hammann F. How far have decision tree models come for data mining in drug discovery? Expert Opin Drug Discov. 2018;13(12):1067-1069.

2. Qi Z, Meng F, Tian Y, et al. Adaboost-LLP: A Boosting Method for Learning With Label Proportions. IEEE Trans Neural Netw Learn Syst. 2018;29(8):3548-3559.

3. Luo Y, Ye W, Zhao X, et al. Classification of Data from Electronic Nose Using Gradient Tree Boosting Algorithm. Sensors (Basel). 2017;17(10).

4. Ogunleye AA, Qing-Guo W. XGBoost Model for Chronic Kidney Disease Diagnosis. IEEE/ACM Trans Comput Biol Bioinform. 2019.

5. Pavey TG, Gilson ND, Gomersall SR, et al. Field evaluation of a random forest activity classifier for wrist-worn accelerometer data. J Sci Med Sport. 2017;20(1):75-80.

6. Nattee C, Khamsemanan N, Lawtrakul L, et al. A novel prediction approach for antimalarial activities of Trimethoprim, Pyrimethamine, and Cycloguanil analogues using extremely randomized trees. J Mol Graph Model. 2017;71:13-27.

7. Maier W, Buller R, Philipp M, et al. The Hamilton Anxiety Scale: reliability, validity and sensitivity to change in anxiety and depressive disorders. J Affect Disord. 1988;14(1):61-68.

8. Manzar MD, BaHammam AS, Hameed UA, et al. Dimensionality of the Pittsburgh Sleep Quality Index: a systematic review. Health Qual Life Outcomes. 2018;16(1):89.

9. Yannuzzi LA. Type-A behavior and central serous chorioretinopathy. Retina. 1987;7(2):111-131
